# Orphan Nuclear Receptor ERRγ Is a Novel Transcriptional Regulator of IL-6 Mediated Hepatic BMP6 Gene Expression in Mice

**DOI:** 10.3390/ijms21197148

**Published:** 2020-09-28

**Authors:** Kamalakannan Radhakrishnan, Yong-Hoon Kim, Yoon Seok Jung, Jina Kim, Don-Kyu Kim, Sung Jin Cho, In-Kyu Lee, Steven Dooley, Chul-Ho Lee, Hueng-Sik Choi

**Affiliations:** 1 School of Biological Sciences and Technology, Chonnam National University, Gwangju 61186, Korea; kmknphd@gmail.com (K.R.); yhemm@naver.com (Y.S.J.); 2 Laboratory Animal Resource Center, Korea Research Institute of Bioscience and Biotechnology, Daejeon 34141, Korea; yhoonkim@kribb.re.kr; 3 Department of Functional Genomics, KRIBB School of Bioscience, University of Science and Technology (UST), Daejeon 34141, Korea; 4 New Drug Development Center, Daegu Gyeongbuk Medical Innovation Foundation, Daegu 41061, Korea; jina@dgmif.re.kr (J.K.); sjcho@dgmif.re.kr (S.J.C.); 5 Department of Integrative Food, Bioscience and Biotechnology, Chonnam National University, Gwangju 61186, Korea; dkkim2@jnu.ac.kr; 6 Leading-Edge Research Center for Drug Discovery and Development for Diabetes and Metabolic Disease, Kyungpook National University Hospital, Daegu 41404, Korea; leei@knu.ac.kr; 7 Department of Internal Medicine, School of Medicine, Kyungpook National University, Kyungpook National University Hospital, Daegu 41944, Korea; 8 Department of Medicine II, Medical Faculty Mannheim, Heidelberg University, 68167 Mannheim, Germany; Steven.Dooley@medma.uni-heidelberg.de

**Keywords:** estrogen-related receptor γ (ERRγ), bone morphogenetic protein 6 (BMP6), interleukin 6 (IL-6), orphan receptors, transcription, gene expression and regulation, liver

## Abstract

Bone morphogenetic protein 6 (BMP6) is a multifunctional growth factor involved in organ development and homeostasis. BMP6 controls expression of the liver hormone, hepcidin, and thereby plays a crucial role in regulating iron homeostasis. BMP6 gene transcriptional regulation in liver is largely unknown, but would be of great help to externally modulate iron load in pathologic conditions. Here, we describe a detailed molecular mechanism of hepatic BMP6 gene expression by an orphan nuclear receptor, estrogen-related receptor γ (ERRγ), in response to the pro-inflammatory cytokine interleukin 6 (IL-6). Recombinant IL-6 treatment increases hepatic ERRγ and BMP6 expression. Overexpression of ERRγ is sufficient to increase BMP6 gene expression in hepatocytes, suggesting that IL-6 is upstream of ERRγ. In line, knock-down of ERRγ in cell lines or a hepatocyte specific knock-out of ERRγ in mice significantly decreases IL-6 mediated BMP6 expression. Promoter studies show that ERRγ directly binds to the ERR response element (ERRE) in the mouse BMP6 gene promoter and positively regulates BMP6 gene transcription in IL-6 treatment conditions, which is further confirmed by ERRE mutated mBMP6-luciferase reporter assays. Finally, an inverse agonist of ERRγ, GSK5182, markedly inhibits IL-6 induced hepatic BMP6 expression in vitro and in vivo. Taken together, these results reveal a novel molecular mechanism on ERRγ mediated transcriptional regulation of hepatic BMP6 gene expression in response to IL-6.

## 1. Introduction

Bone morphogenetic proteins (BMPs) are secreted-type growth factors belonging to the transforming growth factor (TGF)-β superfamily, which also includes TGF-βs, activins and growth differentiation factors [1,2,3]. The BMP subfamily was initially identified as the key proteins involved in cartilage induction and bone formation, later studies demonstrated the importance of BMPs in embryonic development and postnatal tissue remodeling through regulating cell growth, differentiation, chemotaxis and apoptosis [4,5,6,7,8]. Inside the cell, BMPs are synthesized as precursor molecule with hydrophobic secretory leader along with propeptide region joined to the mature region while proteolytic cleavage of precursor protein releases the mature proteins into circulation [9,10]. Within the BMP family, BMP6 is grouped with BMP5, BMP7 and BMP8 based on the carboxy-terminal amino acid (aa) sequence of the mature protein. The mature BMP6 is a disulfide-linked homodimeric protein consists of two 132 aa subunits [11,12]. BMP6 exerts its biological action by binding to the hetero-oligomeric complexes comprising of BMP type I and type II serine-threonine kinase receptors on the cell membrane, which leads to phosphorylated activation of downstream SMAD proteins [13,14,15]. Hepatic BMP6-SMAD signaling plays an important role in iron homeostasis through transcriptionally regulating the liver hormone, hepcidin, which mediates the degradation of iron transporter ferroportin in hepatocytes, macrophages, and enterocytes to control iron entry into the systemic circulation [16,17,18,19].

The estrogen-related receptor γ (ERRγ) along with ERRα and ERRβ constitute the NR3B subfamily of nuclear receptors. ERRs are orphan nuclear receptors, constitutively active without binding to endogenous ligands, which plays diverse biological roles in maintenance of homeostasis and tissue development [20,21,22]. The first cloned orphan nuclear receptors were ERRα and ERRβ which are identified through the search of proteins related to estrogen receptor ERα [23]. ERRγ was recognized through yeast two-hybrid screening, which is the last ERR to be characterized in vivo [24,25]. ERRγ is expressed in several embryonic and adult tissues, especially in tissues with high energy demand such as the heart, brain, kidney, pancreas, and liver. It regulates target gene expression by directly binding to extended half-site core sequences referred to as ERR response element (ERRE) (5′-TCAAGGTCA-3′) as either monomers or dimmers on the regulatory region of the target genes which are primarily involved in cellular metabolism and energy homeostasis [20]. The intrinsic transcriptional activity of ERRγ is predominantly regulated through interaction with co-activator or co-repressor proteins [26,27]. ERRγ plays an important role in glucose, lipids and alcohol metabolism and its hepatic expression is induced in fasting condition [28,29,30,31]. It also involved in iron homeostasis through regulating the expression of hepatic iron regulating hormone, hepcidin [32]. Although ERRγ is constitutively active, it exhibits limited affinity toward many synthetic ligands, including 4-hydroxytamoxifen (4-OHT), which act as an inverse agonist by blocking the binding of ERRγ to response elements on the regulatory regions of target genes [33,34,35].

How hepatocytic BMP6 gene expression is transcriptionally regulated is currently not well understood. As we previously reported the interleukin 6 (IL-6) induced hepatic ERRγ gene up-regulation [32], here we hypothesized that ERRγ may regulate the BMP6 gene expression in hepatocytes. In this study, we demonstrated a novel regulatory mechanism of ERRγ mediated hepatic BMP6 gene expression in response to the pro-inflammatory cytokine, IL-6. Importantly, hepatocyte specific ERRγ knock-out or treatment with an ERRγ specific inverse agonist GSK5182 inhibited IL-6 induced BMP6 gene expression in hepatocytes.

## 2. Results

### 2.1. IL-6 Induces Hepatic ERRγ and BMP6 Gene Expression

**Figure 1:**
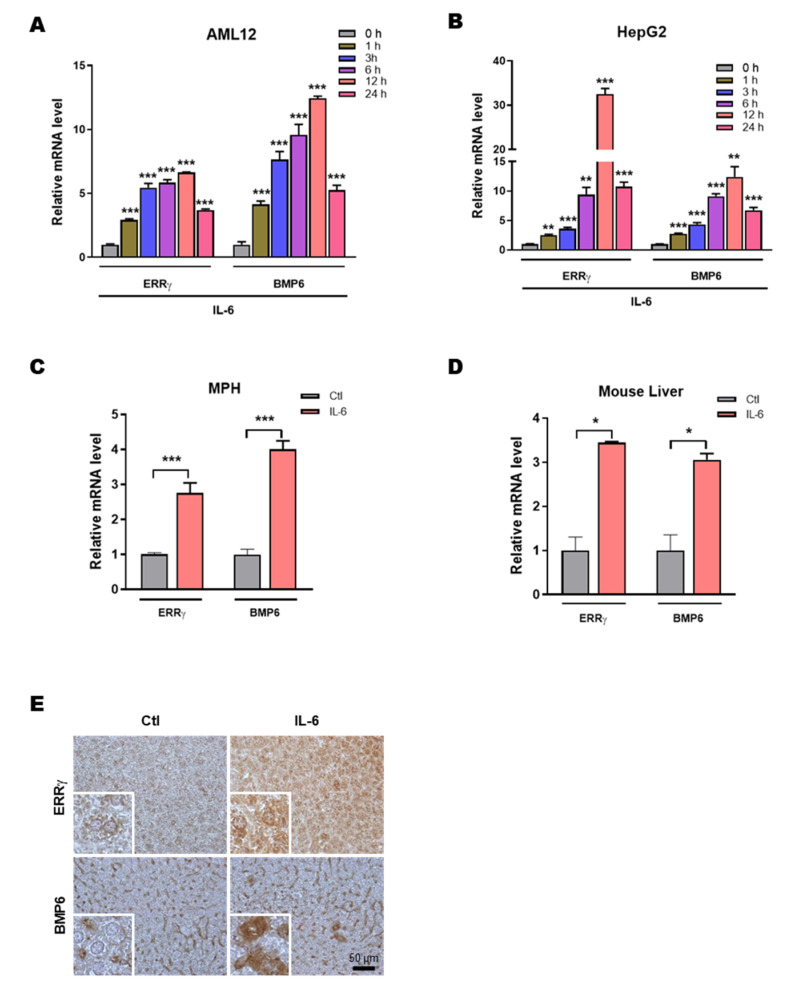
IL-6 promotes hepatic ERRγ and BMP6 gene expression. (**A**,**B**), cultured AML12 (**A**) and HepG2 (**B**) cells were treated with IL-6 for indicated time period. The cells were then harvested for total RNA extraction followed by reverse transcription and quantitative PCR. (**C**) Quantitative PCR analysis of total RNA isolated from cultured mouse primary hepatocytes treated with IL-6 for 12 h. (**D**) Eight-weeks old C57BL6/J mice were intraperitoneally injected with IL-6 (2.5 µg/kg) and sacrificed after 1 h. Livers were harvested for total RNA extraction followed by reverse transcription and quantitative PCR (*n* = 5 mice for control and *n* = 4 mice for IL-6). (**E**) Representative images of ERRγ and BMP6 immunohistochemistry analysis in control or IL-6 treated mice livers. Data are represented as mean + SEM. Asterisks indicate that the difference between the groups was significant, as determined by two-tailed unpaired Student’s *t*-test. * *p* < 0.05, ** *p* < 0.01, *** *p* < 0.001.

In a previous study, we showed that the pro-inflammatory cytokine IL-6 induces ERRγ gene expression in hepatocytes [32]. In the present study, we hypothesized that IL-6 induced hepatic ERRγ may regulate BMP6 gene expression. IL-6 treatment significantly increased ERRγ and BMP6 mRNA expression in AML12 and HepG2 cell lines in a time dependent manner (Figure 1A,B). The result was further confirmed in isolated mouse primary hepatocytes (MPH) treated with IL-6 for 12 h (Figure 1C). To test the effect of IL-6 in vivo, we intraperitoneally injected IL-6 into eight-weeks old C57BL/6J mice and found both ERRγ and BMP6 mRNA levels were significantly increased (Figure 1D). Moreover, through immunohistochemical staining we also found that the protein levels of ERRγ and BMP6 increased in IL-6 treated mouse liver tissues (Figure 1E). These results indicate that IL-6 induces hepatic ERRγ and BMP6 gene expression.

### 2.2. Overexpression of ERRγ Increases BMP6 Gene Expression in Liver

**Figure 2:**
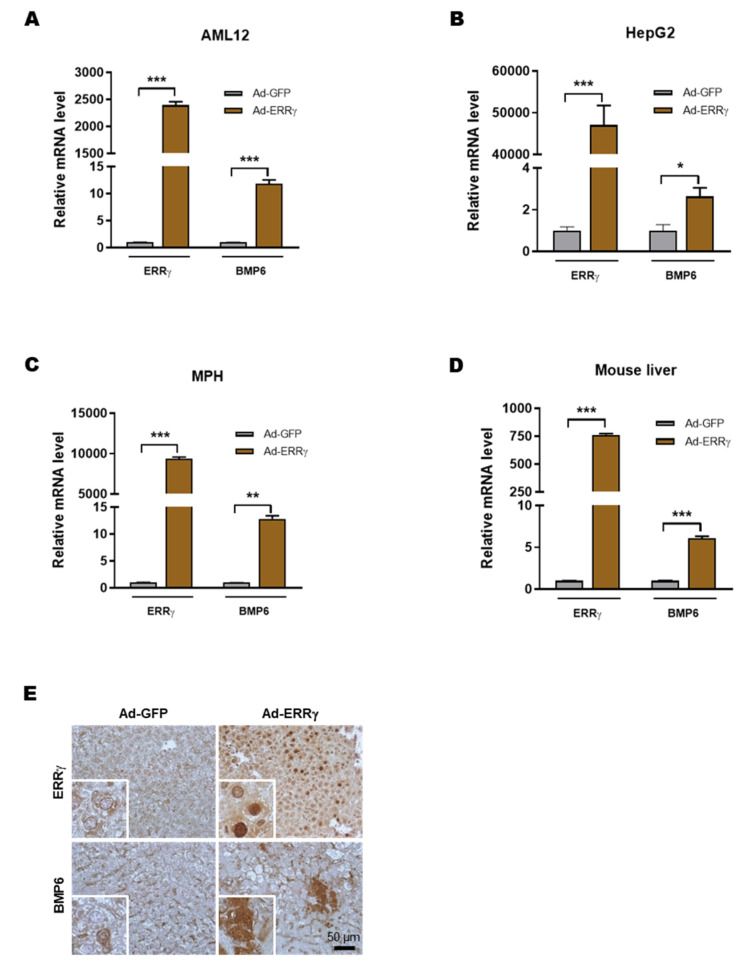
ERRγ increases BMP6 expression in vitro and in vivo. (**A**–**C**) Quantitative PCR analysis showing mRNA levels of ERRγ and BMP6 in AML12 (**A**), HepG2 (**B**) and mouse primary hepatocytes (**C**), infected with Ad-GFP or Ad-Flag-ERRγ for 48 h. **D** and **E**, Either Ad-GFP or Ad-Flag-ERRγ was injected via tail-vein into mice, which were sacrificed at day 5 after injection (*n* = 5 mice per group). Quantitative PCR analysis, showing mRNA levels of ERRγ and BMP6 in mice livers (**D**). Representative image of ERRγ and BMP6 immunohistochemistry analysis in mice livers (**E**). Data are represented as mean + SEM. Asterisks indicate that the difference between the groups was significant, as determined by two-tailed unpaired Student’s *t*-test. * *p* < 0.05, ** *p* < 0.01, *** *p* < 0.001.

Since IL-6 increased both ERRγ and BMP6 gene expression, and ERRγ being a transcription factor, we intended to examine whether ERRγ can regulate hepatic BMP6 gene expression. To test this hypothesis, we overexpressed ERRγ using an adenoviral ERRγ construct (Ad-ERRγ) in AML12, HepG2 and MPH. The BMP6 mRNA level was significantly increased in Ad-ERRγ treated cells compared to controls (Figure 2A–C). This result was verified in C57BL6/J mice by overexpressing ERRγ in hepatocytes through tail-vein injection of Ad-ERRγ. Hepatic BMP6 mRNA level increased in Ad-ERRγ treated mice compared to Ad-GFP treated control mice (Figure 2D). The protein expression of BMP6 in Ad-ERRγ treated mice liver was investigated immunohistochemically and found increased compared to control mice livers (Figure 2E). These results suggest that overexpression of ERRγ is sufficient to induce hepatic BMP6 gene expression.

### 2.3. Liver Specific ERRγ Knock-Out Mice Fail to Increase Hepatic BMP6 Gene Expression in Response to IL-6 Treatment

**Figure 3:**
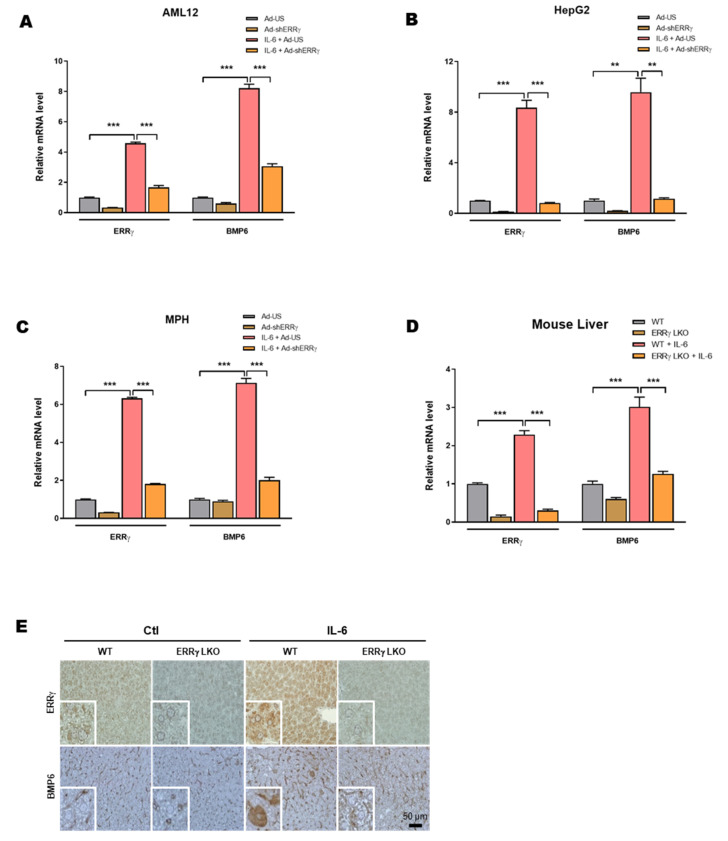
ERRγ deficiency attenuates IL-6 mediated hepatic BMP6 gene expression. (**A**–**C**), Quantitative PCR analysis, showing mRNA levels of ERRγ and BMP6 in AML12 (**A**), HepG2 (**B**) and mouse primary hepatocytes (**C**), infected with an adenovirus expressing an unspecific short hairpin (sh) RNA (Ad-US) or Ad-shERRγ, and treated with IL-6 (20 ng/mL) for 12 h. **D** and **E**, Wild type (WT) or hepatocyte specific ERRγ knock-out (ERRγ LKO) mice were intraperitoneally administered with IL-6 (2.5 µg/kg) and sacrificed (WT and ERRγ-LKO *n* = 5 mice; WT + IL-6 and ERRγ-LKO + IL-6 *n* = 7 mice). Quantitative PCR analysis showing mRNA levels of ERRγ and BMP6 in mice livers (**D**). Representative images of immunohistochemistry analysis of ERRγ and BMP6 in mice livers (**E**). Data represented as mean + SEM. Asterisks indicate that the difference between the groups was significant, as determined by One-Way ANOVA with Tukey’s multiple comparisons test. ** *p* < 0.01, *** *p* < 0.001.

As the above results show that ERRγ overexpression can increase hepatic BMP6 gene expression (Figure 2), we wanted to identify whether ERRγ is required for IL-6 mediated BMP6 gene up-regulation in liver cells. To test this, we knocked-down ERRγ expression in cell lines using adenoviral shERRγ (Ad-shERRγ). IL-6 treatment increased BMP6 mRNA level, whereas Ad-shERRγ significantly blunted IL-6 mediated up-regulation of BMP6 mRNA levels in AML12, HepG2 and MPH (Figure 3A–C). To emphasize the importance of ERRγ in IL-6 mediated hepatic BMP6 gene regulation in vivo, we generated hepatocyte specific ERRγ knock-out mice (ERRγ-LKO). BMP6 mRNA levels were significantly reduced in ERRγ-LKO mice compared to Wild type (WT) mice in response to IL-6 treatment (Figure 3D). Moreover, BMP6 protein expression was also markedly reduced in IL-6 treated ERRγ-LKO mice livers compared to WT mice (Figure 3E). These results imply that ERRγ is important for IL-6 mediated hepatic BMP6 gene up-regulation.

### 2.4. ERRγ Activates the BMP6 Gene Promoter

**Figure 4:**
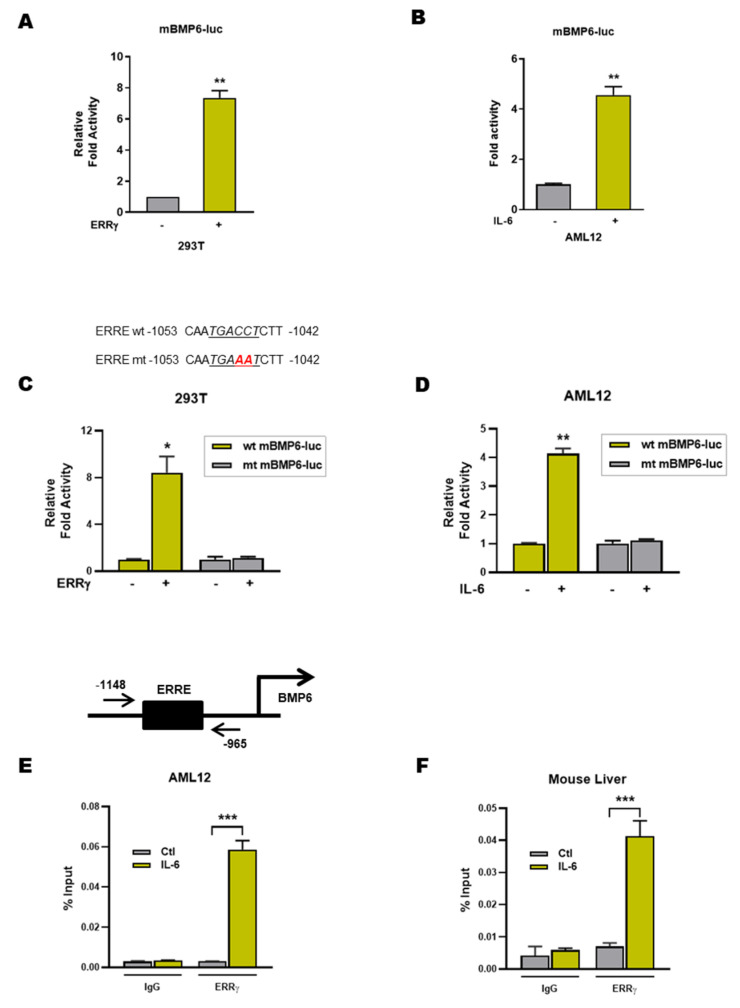
ERRγ activates mouse BMP6 gene promoter activity. (**A**) 293T cells were transfected with a mouse BMP6 promoter-luciferase reporter construct (mBMP6-luc) along with pcDNA3.1 empty vector or pcDNA-ERRγ expression plasmid. (**B**) AML12 cells were transfected with mBMP6-luc and stimulated with IL-6 (20 ng/mL) for 12 h. (**C**) 293T cells were transfected with wild type or ERRE mutant mBMP6-luc along with the ERRγ expression vector. (**D**) AML12 cells were transfected with wild type or ERRE mutant mBMP6-luc and stimulated with IL-6 (20 ng/mL) for 12 h. (**E**) ChIP assay showing binding of ERRγ to the BMP6 gene promoter. AML12 cells were treated with IL-6 and soluble chromatin was immuno-precipitated with an ERRγ antibody. Purified DNA samples were used for quantitative PCR with primers corresponding to the ERRE region of the mouse BMP6 gene promoter. (**F**) In vivo ChIP assay, showing binding of ERRγ to the BMP6 gene promoter in response to IL-6. We treated C57BL6/J mice with IL-6 and immuno-precipitated soluble chromatin from mice livers with an ERRγ antibody. Purified DNA was used for quantitative PCR analysis with primers corresponding to the ERRE region of the mouse BMP6 gene promoter. Data are represented as mean + SEM. Asterisks indicate that the difference between the groups was significant, as determined by two-tailed unpaired Student’s *t*-test. * *p* < 0.05, ** *p* < 0.01, *** *p* < 0.001.

We next examined the potential molecular mechanism underlying ERRγ mediated BMP6 gene up-regulation in response to IL-6 treatment. To explore the mechanism, we first cloned the mouse BMP6 gene promoter into a luciferase reporter construct and transiently transfected 293T and AML12 cells. Co-transfection with an ERRγ expression vector or IL-6 treatment significantly increased BMP6 promoter activity in both cell types (Figure 4A,B). A thorough investigation of the mouse BMP6 promoter revealed a putative ERRγ binding motif, ERRE, at 1045 bp upstream of the transcription start site. To verify the relevance of this putative ERRγ binding site, we constructed an ERRE mutant mouse BMP6 promoter-luciferase reporter, and found that promoter activation by ERRγ overexpression was significantly abolished (Figure 4C). Moreover, the observed IL-6 induced 4-fold increase of BMP6 gene promoter activity in the wild type construct was absent when using the ERRE mutant promoter construct as readout (Figure 4D). Finally, binding of ERRγ to the endogenous genomic BMP6 promoter was confirmed by chromatin immuno-precipitation (ChIP) assay, using ERRγ specific antibody precipitation in AML12 cells that were treated with IL-6 (Figure 4E). To further validate this result in vivo, we performed comparative ChIP assays in control and IL-6 treated mouse liver tissues, which showed that ERRγ was recruited to the ERRE region of the BMP6 gene promoter in response to IL-6 treatment (Figure 4F). In total, our results indicate that ERRγ regulated BMP6 gene transcription occurs by direct binding and activating the BMP6 gene promoter.

### 2.5. An Inverse Agonist of ERRγ Inhibits IL-6 Induced Hepatic BMP6 Gene Expression

**Figure 5:**
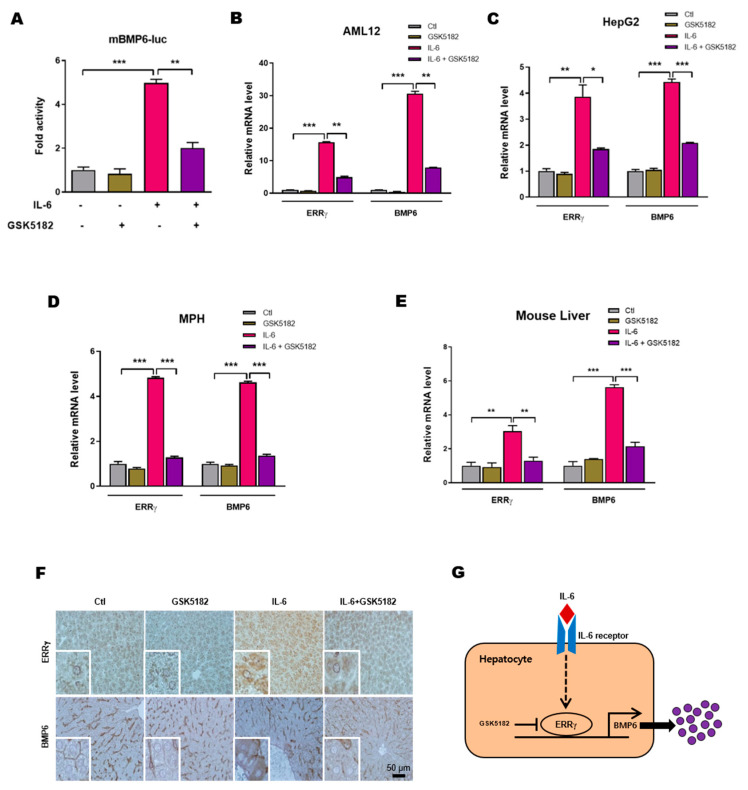
GSK5182 inhibits IL-6 mediated hepatic BMP6 gene expression. (**A**) AML12 cells were transfected with mBMP6-luc and stimulated with IL-6 (20 ng/mL) for 12 h, in presence or absence of GSK5182 (10 µM). (**B**–**D**) Quantitative PCR analysis, showing mRNA levels of ERRγ and BMP6 in AML12 (**B**), HepG2 (**C**) and mouse primary hepatocytes (**D**), treated with IL-6 (20 ng/mL) for 12 h, in presence or absence of GSK5182 (10 µM). **E** and **F**, Mice were intraperitoneally injected with IL-6 (2.5 µg/kg), with or without GSK5182 (40 mg/kg) and sacrificed (control and GSK5182 *n* = 4 mice; IL-6 and GSK5182+IL-6 *n* = 6 mice). Quantitative PCR analysis showing mRNA levels of ERRγ and BMP6 in mice livers (**E**). Representative image of ERRγ and BMP6 immunohistochemistry analysis in mice livers (**F**). (**G**) A schematic model of IL-6 induced ERRγ mediated BMP6 production from hepatocytes. Data are represented as mean + SEM. Asterisks indicate that the difference between the groups was significant, as determined by One-Way ANOVA with Tukey’s multiple comparisons test. * *p* < 0.05, ** *p* < 0.01, *** *p* < 0.001.

The above results provide strong evidence that ERRγ is important for BMP6 gene up-regulation in hepatocytes in response to IL-6 treatment, so we intended to test the effect of pharmacological inhibition of ERRγ on BMP6 expression. GSK5182, a 4-OHT analog that acts as a specific inverse agonist of ERRγ by blocking the binding of ERRγ to ERRE motifs on target gene promoters, and thereby inhibiting ERRγ transactivation [30,32]. In AML12 cells, IL-6 mediated BMP6 gene promoter activity was significantly reduced by GSK5182 (Figure 5A). Furthermore, IL-6 induced BMP6 mRNA up-regulation was markedly inhibited by GSK5182 in AML12, HepG2 and MPH (Figure 5B–D). Next, we assessed the effect of GSK5182 in vivo by intraperitoneally injecting mice with IL-6 in presence or absence GSK5182. BMP6 mRNA level increased in IL-6 treated mouse livers, whereas GSK5182 significantly inhibited IL-6 induced BMP6 mRNA expression (Figure 5E). Consistent with mRNA levels, IL-6 induced hepatic BMP6 protein levels were also markedly reduced by GSK5182 treatment, as investigated by immunohistochemistry (Figure 5F). Taken together, these results suggest that inverse agonist mediated inactivation of ERRγ transcriptional activity significantly inhibits IL-6 induced hepatic BMP6 gene up-regulation.

## 3. Discussion

Our study revealed a novel transcriptional regulatory mechanism of BMP6 gene expression in hepatocytes by an orphan nuclear receptor ERRγ in response to an inflammatory cytokine, IL-6 treatment (Figure 5G). Overexpression of ERRγ is sufficient to induce BMP6 gene expression in hepatocytes, whereas loss of hepatic ERRγ expression significantly inhibited IL-6 mediated BMP6 up-regulation. Moreover, GSK5182, an inverse agonist of ERRγ showed effective inhibitory action toward BMP6 gene expression in IL-6 treated mice liver.

Several reports imply the importance of BMP6 in iron homeostasis through endogenously regulating the expression of hepcidin, a hormone mainly produced by hepatocytes that controls the entry of iron into plasma [18,19]. However, the exact mechanism underlying the regulation of BMP6 gene expression in liver is largely unknown. In a recent study, the transcription factor nuclear factor erythroid 2-related factor 2 (Nrf2) was reported to regulate hepatic BMP6 expression in response to iron by directly binding the BMP6 gene promoter [36]. Here, we show that orphan nuclear receptor ERRγ transcriptionally regulates hepatic BMP6 expression by directly binding the ERRE motif in the BMP6 gene promoter, in response to IL-6 treatment. We found a functional ERRE motif AGGTCA in the BMP6 gene promoter 1045 bp upstream of the transcription start site, and an ERRE mutant BMP6 promoter showed a significantly blunted response to endogenous/exogenous ERRγ mediated promoter activation. Baseline BMP6 expression was not affected by mutating the ERRE; however, under inducible conditions, such as in IL-6 treatment, the ERRE mutation significantly blunted BMP6 expression. Thus, ERRγ acts a novel transcriptional regulator of IL-6 mediated hepatic BMP6 gene expression. Previous reports suggest that IL-6 and BMP signaling synergistically may regulate hepatic hepcidin gene expression, and that inhibition of BMP signaling attenuates IL-6 induced hepcidin expression [37]. We now describe ERRγ as mediator of the IL-6 mediated hepatic BMP6 expression, suggesting existence of an autocrine BMP6 signaling loop to up-regulate hepcidin gene expression in hepatocytes.

The liver consists of different cell types, which are divided into parenchymal and non-parenchymal cells (NPCs). Parenchymal cells encompass hepatocytes, which is the major cell type in liver and to a minor extent cholangiocytes. NPCs include hepatic stellate cells, Kupffer cells and liver sinusoidal epithelial cells. Hepatic BMP6 production was reported from NPCs in several reports [36,38,39]. In particular, it was reported that BMP6 basal expression was confined to NPCs, namely HSCs and KCs, and treatment with TGF-β1 increased BMP6 gene expression in HSCs [40]. However, hepatocyte derived BMP6 production was also reported [41,42]. Murine hepatocytes were reported to produce BMP6 in an in vitro model of cellular lipid accumulation and hepatocytes were identified as cellular source of hepatic BMP6 in livers of patients with non-alcoholic fatty liver disease [41]. Chronic dietary iron changes are reported to modulate liver BMP6 expression in all cell types, including hepatocytes [42]. In the present study, we have confirmed by quantitative PCR and immunohistochemistry that hepatocytes produce BMP6 in IL-6 treated mice livers through transcriptional activation by ERRγ. Hepatocyte specific ERRγ knock-out mice significantly inhibited IL-6 induced hepatic BMP6 expression. Moreover, a small molecule GSK5182 mediated inhibition of ERRγ transactivation markedly reduced hepatic BMP6 expression in IL-6 treated mice. Taken together, in livers of IL-6 injected mice, hepatocytes are the source of BMP6, which is transcriptionally regulated by ERRγ. The proposed IL-6-ERRγ-BMP6 pathway is probably active in inflammatory conditions when pro-inflammatory cytokines, including IL-6, levels are increased and may up-regulate hepcidin expression, leading to hypoferremia and anemia, such as in inflammatory anemia.

In conclusion, we report that ERRγ plays an important role in IL-6 mediated BMP6 gene expression in vivo. GSK5182, an ERRγ inverse agonist significantly inhibits ERRγ-dependent BMP6 gene expression. Therefore, we suggest ERRγ as a novel transcriptional regulator of hepatic BMP6 expression.

## 4. Materials and Methods

### 4.1. Animal Experiments

Eight to twelve-week-old male C57BL/6J mice were used for all experiments. C57BL/6J wild type (WT) mice were obtained from Korea Research Institute of Biosciences and Biotechnology (KRIBB, Daejeon, Korea). C57BL/6J mice containing floxed ERRγ exon 2 (ERRγf/f) were obtained from PHENOMIN-iCS, PHENOMIN, the French National Infrastructure in Biology and Health (Illkirch, France). To produce the liver specific ERRγ knockout line (ERRγ-LKO), ERRγf/f animals were crossbred with C57BL/6J-Alb-Cre transgenic mice, which express Cre recombinase in hepatocytes under the control of the albumin promoter (Jackson Laboratories, Bar Harbor, ME, USA). Prior to the experiments, mice were acclimatized to a 12-h light/dark cycle at 22 ± 2 °C for 2 weeks with unlimited food and water in a specific pathogen-free facility. WT mice were injected with Ad-GFP or Ad-FLAG-ERRγ via tail-vein for 5 days. WT and ERRγ-LKO mice were treated with 2.5 µg/kg of recombinant human IL-6 via intraperitoneal (i.p) injection for indicated time period. Where indicated, GSK5182 (40 mg/kg in 30% PEG400) was administered intraperitoneally (i.p.) into mice. All animal procedures were approved by the Chonnam National University Animal Care and Use Committee (CNU-IACUC-YB-2017-40) and by the Institutional Animal Care and Use Committee of KRIBB (KRIBB-AEC-19128). All animal experiments were performed in accordance with the Guide for the Care and Use of Laboratory Animals published by the US National Institutes of Health.

### 4.2. Cell Culture, Transient Transfection and Luciferase Assay

AML12 (mouse immortalized hepatocytes), HepG2 (human hepatoma cells) and 293T (human embryonic kidney cells) were obtained from the American Type Culture Collection (Manassas, VA, USA). All cell lines were maintained as described previously [43]. Transient transfections were performed using Lipofectamine 2000 (Invitrogen, Carlsbad, CA, USA) and FuGENE HD (Promega, Madison, WA, USA) transfection reagents according to the manufacturer’s instructions. Luciferase assay carried out as described previously [43]. Briefly, cells were transfected with indicated reporter plasmids together with expression vectors encoding ERRγ or treated with IL-6. Total cDNA used for each transfection was adjusted to 1 mg/well by adding an appropriate amount of empty vector and pCMV–β-gal plasmid was used as an internal control. The luciferase activity was normalized to β-galactosidase activity.

### 4.3. Isolation of Primary Hepatocytes

Primary hepatocytes were isolated from C57BL/6J mice by collagenase perfusion [44] and seeded with Dulbecco’s modified Eagle’s medium (DMEM) (Gibco-BRL, Grand Island, NY, USA) supplemented with 10% fetal bovine serum (Hyclone, Logan, UT, USA) and antibiotics in a humidified atmosphere containing 5% CO_2_ at 37 °C. After attachment, cells are infected with adenovirus (Ad-GFP, Ad-ERRγ or Ad-shERRγ) or treated with IL-6 for the indicated time period.

### 4.4. Chemicals and Antibodies

Recombinant human IL-6 is obtained from Prospec Protein Specialists (East Brunswick, NJ, USA, Cat#cyt-213), and GSK5182 was synthesized as previously described [28,34,35,45]. For immunohistochemical staining of endogenous ERRγ, rabbit anti-ERRγ serum was generated using a peptide (404-AGQHMEDPRRAGKMLM-419) from mouse ERRγ helix 9 [46] (AbFrontier/Young in Frontier, Seoul, Korea). Antibody was affinity purified using the same peptide and tested by Western blotting. No cross reactivity to ERRα or ERRβ was noted [47]. Mouse monoclonal anti-BMP6 antibody (Abcam, Cambridge, MA, USA, Clone#morph-6.1, Cat#ab15640) was used for immunohistochemical staining of BMP6.

### 4.5. DNA Cloning

Mouse BMP6 gene promoter (−1.5 kb) was PCR-amplified from mouse genomic DNA (Promega) using the primer: forward, 5′-TCAACGACACCACAAAGAGTTC-3′ and reverse, 5′-TGCAAGACTTGGTAAATGCTGA-3′ and inserted into the pGL3 basic vector (Promega) using the NheI and XhoI restriction enzyme sites. ERRE mutant constructs were generated using QuikChange Lightning Site-Directed mutagenesis kit (Agilent Technologies, Palo Alto, CA, USA, Cat#210518). Ad-GFP and Ad-FLAG-ERRγ were described previously [32]. The vector expressing ERRγ was described previously [31].

### 4.6. Quantitative PCR

Total RNA was isolated from cell lines, mouse primary hepatocytes or mice liver tissues using the TRIzol reagent (Invitrogen), according to the manufacturer’s instructions. cDNAs generated by Top Script cDNA synthesis kit (Enzynomics, Daejeon, Korea) were analyzed by the Applied Biosystems StepOnePlus real-time PCR system (Applied Biosystems) using Power SYBR Green PCR Master Mix (Applied Biosystems, Foster City, CA, USA). All data were normalized to ribosomal protein L32 expression. Primers used in this study: Mouse ERRγ, forward 5′-AAGATCGACACATTGATTCCAGC-3′ and reverse, 5′-CATGGTTGAACTGTAACTCCCAC-3′; Mouse BMP6, forward 5′-TCAACGACACCACAAAGAGTTC-3′ and reverse, 5′-TGCAAGACTTGGTAAATGCTGA-3′; and mouse L32, forward 5′-GTGAAGCCCAAGATCGTCAA-3′ and reverse, 5′-TGTCAATGCCTCTGGGTTTC-3′.

### 4.7. Chromatin Immunoprecipitation (ChIP) Assay

Formaldehyde cross-linking and chromatin immuno-precipitation (ChIP) analyses were performed as previously described [29] using SimpleChIP Plus Enzymatic Chromatin IP kit (Cell Signaling Technology, Danvers, MA, USA, Cat log#25268S) according to manufacturer’s protocol. Mouse monoclonal anti-ERRγ antibody (Perseus Proteomics, Tokyo, Japan, Clone#H6812, Cat#PP-H6812-00) was used for immunoprecipitation. The following primer was used for real-time quantitative PCR analysis of ChIP eluted and Input DNA. Mouse BMP6 ERRE site, forward 5′-CGGTAACGGCATGGATTAAATAG-3′ and reverse 5′-TGCCTAGCGAGCGTAGGTTTCTA-3′.

### 4.8. Immunohistochemistry

Liver samples were fixed in 10% neutral buffered formalin, embedded in paraffin, cut into 5 μm-thick sections. To detect ERRγ and BMP6 protein, liver sections were stained with an anti-ERRγ and anti-BMP6 antibody and visualized using 3,3′-diaminobenzidine (Vector Lab. Inc., Burlingame, CA, USA). Images were captured using a light microscope (BX51, Olympus Corporation, Tokyo, Japan).

### 4.9. Statistical Analysis

Data were analyzed using Prism 8 (GraphPad Software, La Jolla, CA, USA). Data are expressed as mean + SEM. The two-tailed Student’s *t*-test was used for statistical analysis between two groups, whereas statistical significance between multiple treatment groups was determined by analysis of one-way ANOVA with Tukey’s multiple comparisons test. Differences were considered statistically significant at *p* < 0.05.

## Abbreviations

BMP6	Bone morphogenetic protein 6
ChIP	Chromatin immunoprecipitation
ERRγ	Estrogen-related receptor γ
ERRE	ERR responsive element
IL-6	Interleukin 6
NPCs	Non-parenchymal cells
4-OHT	4-hydroxytamoxifen
